# Q and A with Outgoing ASTMH President Joel Breman

**DOI:** 10.4269/ajtmh.20-interview

**Published:** 2021-01-15

**Affiliations:** Joel Breman, MD, DTPH, FASTMH, Scientist Emeritus with the Fogarty International Center at NIH, recently completed his term as ASTMH president. Breman is internationally known for his work on Ebola, smallpox, malaria, and other diseases. He was part of the team that in 1976 investigated the first known outbreak of Ebola virus disease, and also served as deputy chief of the WHO’s Smallpox Eradication Unit. He recently sat down with science writer Matthew Davis to discuss the challenges of leading the Society during the COVID-19 pandemic and the lessons from this tumultuous year in global health.

## In your President’s Address at the Annual Meeting, you described 2020 as a “year like no other.” For an infectious disease veteran—someone who was deeply involved in the fight against smallpox and Ebola—what has felt familiar about the COVID-19 pandemic and what has felt very different from past events?

For many years, when you would talk to people in our field about their biggest concerns, their fears, and their bad dreams, it involved a global outbreak of a severe respiratory disease. Typically, they would think about influenza. But the fact that this has been a pandemic that involved a respiratory disease felt familiar or at least not unexpected. We had seen the first SARS virus come out of this same region years ago.

**Figure:**
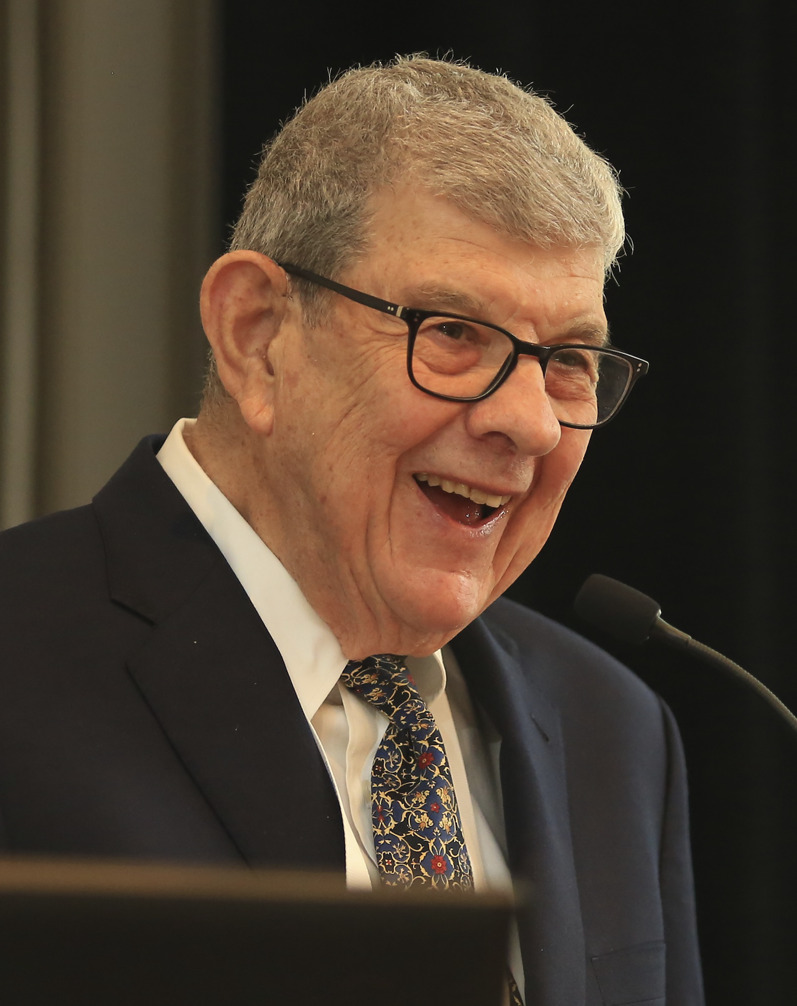
**2020 President Joel G. Breman, MD, DTPH, FASTMH**

We also had, very early in this situation, a lot of sharing of genetic and epidemiological information, and that was also something I had anticipated happening.

But then, there were different, unexpected things that happened. There was the fact that so many countries, including wealthy countries, were very quickly overwhelmed by this virus. And there also was a lot of confusion. The leadership in the United States was especially chaotic. There seemed to be a shortage of just about everything, and it seemed like the worst possible choice to leave it to states and local communities to deal with it on their own. That was both very depressing and very different from what I expected. Because when emergencies occur, people usually pull together. That did not happen.

## There has been a strong, often divisive political dimension to the pandemic that has made it all the more challenging. How did the Society find its voice in such a charged environment?

Our organization is one focused on poor countries so that was our number one priority early in the pandemic. We wanted to illuminate the problems in Africa, Latin America, and India. We used our journal, the *American Journal of Tropical Medicine and Hygiene*, to publish insights into the situation in different countries and regions. We also wanted to strongly support the WHO in its work. And as a science organization, we focused on providing evidence in regard to treatment and prevention. For example, we looked into the use of unproven interventions, like hydroxychloroquine.

But we also were outraged by the fact that China was almost immediately accused of fomenting this pandemic and by the attacks on the WHO. So we published a detailed scientific analysis of the origins of SARS-COV2 in the Journal and developed a related commentary for STAT. We also published an editorial that forcefully protested politically-motivated decisions to cut funding for a coronavirus research project that included Chinese colleagues and to terminate the United States’ relationship with the WHO. 

## What can we take away from this pandemic that could be constructive going forward for addressing global health challenges, both in terms of strategies and tools and innovations?

A lot of people will be asking: “How can we do better next time?” And it always comes back to what I call the five Ps: preparation, performance, partnerships, prediction/prevention, and, finally, practice—things like tabletop exercises that simulate a crisis.

In terms of tools and innovations, we have seen an interesting contrast.

I remember early in the outbreak I called a researcher at a very well-known institution to see if he could get involved in the Society’s response. And he said “I can’t do it. I’m overwhelmed with patients and I’m scrambling just to find nasal swabs to take specimens.” Around the same time, I was writing a chapter on smallpox with a coauthor at the NIH. And 1 day, he called to say he no longer had time for the project because the SARS-CoV-2 genome had just been posted on the Internet and he had to start working on diagnostics and other new products, which included the Moderna vaccine.

So we have seen these extremes during this pandemic—the ability to rapidly innovate and very quickly develop new vaccines, while at the same time, we have been hobbled by a lack of the most basic tools.

## Was it surprising to see your friend Anthony Fauci become an international media celebrity?

Dr. Fauci has been doing this for several decades, and he has learned through hard experience how to be a very good communicator. He is clear and understandable, and he speaks the truth. He also knows how to speak to different audiences. He is comfortable talking with scientists, to the media, and sitting down with people like (Washington Nationals baseball player) Ryan Zimmerman and actress Julia Roberts. He was fearless in the face of despicable attacks and threats. And he was physically impressive. At one point, he had to have a polyp removed from his vocal chords, and then a few days later, he was back in action.

## What should be the Society’s priorities when it comes to crafting a post-pandemic agenda for 2021 and beyond?

We have traditionally been focused on infectious diseases, but we are realizing the importance of a broader focus. That includes paying more attention to the growing threat of noncommunicable diseases in poor countries, such as cardiac disease and cancer. As a friend of mine says, “if you live like people in the west, you will die like people in the west.” I think mental illness also deserves more attention. And we see the Society taking more interest in many broader challenges that have a significant impact on health, like gender discrimination, social justice concerns, and climate change.

## In the United States, the arrival of the Biden administration means new leadership in the United States and new people heading key agencies and programs. What should the Society be doing in terms of outreach?

We have to remember that we are an apolitical and nonpartisan Society. But we should not be passive, and we should be available to assist the new administration. We already are involved with like-minded organizations in advocating for the health needs of low-income and middle-income countries. And we should continue to inform the decision-making at key agencies and seek more resources to support their research programs. We also will continue to show our support for the WHO and Dr. Tedros.

We can have more influence in Washington if our members focus on local outreach—writing an op-ed in the local paper and speaking to their representative in Congress. We need more people to understand that much of our work is happening in their local communities, such as in university research departments. That can help decision-makers in Congress feel a stronger connection to our priorities

